# Cost-effectiveness of treating hepatitis C in Seychelles

**DOI:** 10.11604/pamj.2019.33.26.17742

**Published:** 2019-05-15

**Authors:** Naomi Joan Faray Adeline, Claudia Geue, Mohsen Rezaei Hermami

**Affiliations:** 1 Communicable Disease Control Unit, Ministry of Health Seychelles, Victoria, Seychelles; 2 Health Economics and Health Technology Assessment, University of Glasgow, United Kingdom; 3 PenTAG Health Technology Assessment, University of Exeter, United Kingdom

**Keywords:** Hepatitis C, direct-acting antiviral, cost-effectiveness

## Abstract

**Introduction:**

approximately eighty million people around the world are living with hepatitis C, and 700,000 people die every year, due to hepatitis C related complications. In Seychelles, a total of 777 cases of hepatitis C were reported from 2002 to 2016, but up to mid of 2016, the cases were not being treated. Treatment with Harvoni, a combination of sofosbuvir and ledipasvir (SOF/LDV), is now being offered on the condition that the patient does not, or has stopped, injecting drugs. This paper is the first to establish the cost effectiveness of treating all cases of hepatitis C in Seychelles with Harvoni, as compared to no treatment.

**Methods:**

data extracted from literature was used to populate an economic model to calculate cost-effectiveness from Seychelles' government perspective. The model structure was also informed by the systematic review and an accompanying grading of economic models using the Consolidated Health Economic Evaluation Reporting Standard (CHEERS) checklist. A Markov model was developed, employing a lifetime horizon and costs and benefits were analysed from a payer's perspective and combined into incremental cost effectiveness ratios (ICERs).

**Results:**

the direct-acting antiviral (DAA), Harvoni, was found to be cost-saving in Seychelles hepatitis C virus (HCV) cohort, as compared to no treatment, with an ICER of € 753.65/QALY. The treatment was also cost-saving when stratified by gender, with the ICER of male and female being € 783.74/QALY and € 635.20/QALY, respectively. Moreover, the results obtained from acceptability curves showed that treating patients with Harvoni is the most cost-effective option, even for low thresholds.

**Conclusion:**

treating hepatitis C cases in Seychelles is cost-saving. It is worth developing a treatment programme to include all cases of hepatitis C, regardless of status of drug injection.

## Introduction

The World Health Organization estimates that over 80 million people worldwide are living with chronic infection of hepatitis C (HCV), with a global prevalence of 1.1% [1]. Every year, more than 700,000 deaths occur due to complications related to hepatitis C, such as liver cirrhosis, liver failure and hepatocellular carcinoma [1]. Up to 2014, the mainstay of hepatitis C treatment in high income countries was weekly PEGylated interferon for 24-28 weeks, along with ribavirin for about six months [1]. The cure rates were less than 50%, the regimen was expensive, and there were significant side effects, such as headaches, muscle aches and fever [1]. The first all oral HCV treatment, direct-acting antivirals (DAAs), were approved by the European commission in January 2014 [2]. The side effects of DAAs are minimal, and the cure rates are more than 90% after 12 weeks of treatment, regardless of co-morbidities or stage of disease of the patient [1]. Several countries have now updated their treatment programmes [1]. Despite this revolution in the treatment, hepatitis C elimination still faces several barriers. Firstly, the cost of the DAAs are high for both individuals and health systems [1]. Secondly, the access to treatment is lower than ideal for those who are known to be at higher risk of getting infected, such as people who inject drugs (PWID) [3]. PWID are a high-risk group due to the transmission of hepatitis C virus through sharing of needles during drug use [4]. DAAs are now recognised as the recommended treatment for hepatitis C worldwide [5]. The World Health Organization (WHO) has released updated screening and treatment guidelines to assist countries in making decisions while developing their treatment protocols [5]. In May 2016, the World Health Assembly (WHA) adopted a strategy to reduce hepatitis C incidence by 90% and reduce mortality by 65% by 2030 [6]. To achieve these targets, prevention and treatment programmes will have to be scaled up [1, 6]. In the context of treatment, the requirement is to place 80% of patients with chronic hepatitis C on treatment [1, 6]. However, the barriers, as mentioned earlier, need to be addressed.

**Figure 1:**
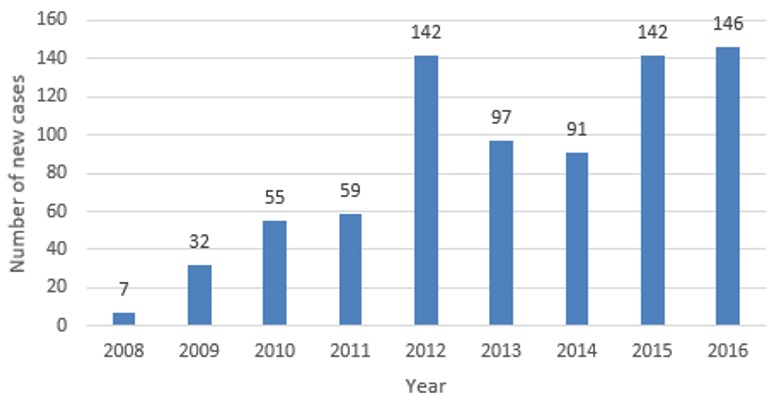
new hepatitis C cases from 2008 to 2016 in Seychelles

Seychelles is a group of islands in the Indian Ocean, just off the east coast of mainland Africa. It had a total estimated population of 94,205 at the end of 2016, the smallest of any African nation [7]. The country has universal healthcare that is publicly funded for all citizens [8]. Under the umbrella of the Public Health Agency (PHA), the Communicable Disease Control Unit (CDCU) is the only Specialist Referral Centre (SRC) in Seychelles for management of communicable diseases, including hepatitis C [8]. The first positive diagnostic test result for hepatitis C in Seychelles was in 2002 [9]. Between 2002 and 2016, a total of 777 cases were reported, of which 648 cases (83%) were males and 129 cases (17%) were females [9]. The key population group most at risk are people who inject drugs (PWID), making up 99% of the hepatitis C cases recorded [9]. Furthermore, 16% were co-infected with both hepatitis C and HIV [9]. In this same time frame, there were 26 (3%) hepatitis C related deaths, of which 16 were males and 10 were females [9]. Figure 1 shows the increasing trend in hepatitis C incidence in Seychelles, from 2008 to 2016. The DAA Harvoni was introduced into the hepatitis C treatment options as of June 2016 [10]. Thirty patients have initiated treatment with it in the first year [10]. Harvoni is a combination of sofosbuvir and ledipasvir (SOF/LDV), produced by Gilead Sciences [1]. It is now the recommended treatment for HCV infection with genotypes 1, 4, 5 and 6 [1, 5]. Seychelles has access to Harvoni, at the cost of $800 for 8 weeks course, and $1200 for 12 weeks course, for each patient [10]. The current recommended duration of treatment for Harvoni is 12 weeks, regardless of viral load [5]. Treatment provision in Seychelles is prioritised for those who have stopped, injecting drugs [10]. Persons still injecting drugs continue to have limited access to treatment for hepatitis C [10]. Their high risk of re-infection together with the high cost of the current treatment plays a major role here [10]. We aimed to evaluate the cost effectiveness of treating all the hepatitis C cases in the Seychelles population, in comparison to not treating these cases.

## Methods

**Figure 2:**
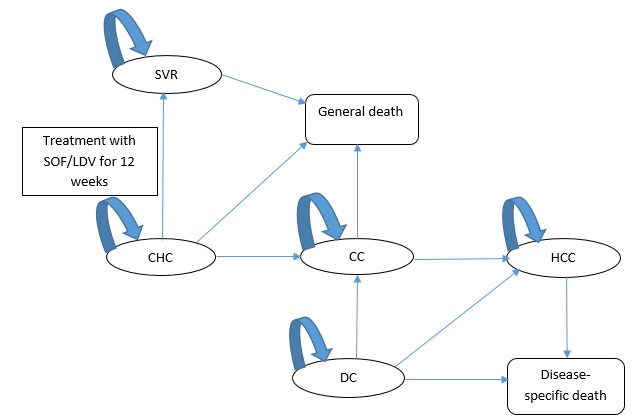
diagram of Markov model. The health states are chronic hepatitis C (CHC), sustained virologic response (SVR), compensated cirrhosis (CC), death from general mortality (General death), decompensated cirrhosis (DC), hepatocellular carcinoma (HCC), and death due to complications of hepatitis C (Disease-specific death). The arrows indicate the direction of possible transitions from one state to another

A systematic review was carried out, using MEDLINE (Ovid), Embase (Ovid) and University of York Centre for Reviews and Dissemination, and data was extracted from the 21 identified studies. Transition probabilities and utility values used were extracted from literature. The EuroQol 5 dimensions questionnaire was used to report utilities. Evidence from the systematic review was used to populate an economic model to calculate cost-effectiveness from Seychelles' Government perspective. The model structure was also informed by the systematic review and an accompanying grading of economic models using the Consolidated Health Economic Evaluation Reporting Standard (CHEERS) checklist. The economic model used was a Markov model, accompanied by a lifetime horizon and a payer's perspective, as shown in Figure 2. Outcome measures included quality adjusted life years (QALYs) and incremental cost effectiveness ratios (ICERs). Two simulated cohorts, a treatment cohort (Rx cohort) and a control cohort (no Rx cohort), of 1000 HCV patients each, were followed through the Markov model for a duration of 60 years. The cohort was representative of the Seychelles cohort of patients infected with hepatitis C by the end of 2016, with the mean age of the patients being 32 years, 84% being male and 16% being female [11].

## Results

**Base case analysis**: the results showed that treating HCV patients with Harvoni (SOF/LDV) was cost-saving as compared to no treatment. This is presented in Table 1 below. Treatment was seen to have both, a lower cost and higher effectiveness than no treatment, consequently dominating the latter. The cost of treatment of all hepatitis C cases in Seychelles with DAAs over a period of 60 years was estimated at € 2,025,834.92, compared to current care protocols, which are estimated to cost € 14,702,231.13. Thus, saving the payer an estimated € 12,676,396.21, if DAAs are introduced. The effect of treatment was 31,758.23 quality adjusted life years (QALYs), compared to just 14,938.19 QALYs for the no treatment option. Therefore, treatment is preferred, with a gain of 16,820.04 QALYs over 60 years. The incremental cost-effectiveness ratio is € 753.65/QALY, with dominance of the treatment option over the no treatment option. The latter was both more expensive and less effective. When discounting of costs is not applied, both the costs of treatment and no treatment cohorts are higher. This is presented in Table 2 below. Furthermore, the cost gap between the two treatment options is raised from € 12,676,396.21 to € 25,167,920.84. However, these results are presented here for comparison sake only. Table 3 and Table 4 below show results for male and female subgroups, respectively. The difference in results of the two subgroups were driven by the gender differences in general mortality rate. Stratification by gender results in the same conclusion of treatment with Harvoni being cost saving, as compared to the no treatment option, with negative ICER results for both subgroups. However, the results show that it is more cost-saving to treat male patients than it is to treat female patients, reflected in the lower ICER for male group than for female group. This may be explained by the lower life expectancy in males for all age-groups.

**Table 1:** results of Markov Simulation Model (MSM) comparing cost-effectiveness of Harvoni compared to no treatment for all HCV patients in Seychelles

Comparator	Cost (€)	QALY	Incremental cost (€)	Incremental QALY	ICER (cost/QALY)	Subset
Rx cohort	2,025,834.92	31,758.23				Dominant
No Rx cohort	14,702,231.13	14,938.19	-12,676,396.21	16,820.04	-753.65	Dominated

**Table 2:** comparison of results for undiscounted and discounted costs

	Undiscounted costs (€)	Discounted costs (€)
Rx cohort	2,205,770.92	2,025,834.92
No Rx cohort	25,167,920.84	14,702,231.13
Incremental costs	-22,962,149.92	-12,676,396.21

**Table 3:** results of Markov Simulation Model comparing cost-effectiveness of Harvoni compared to no treatment for male HCV patients in Seychelles

Comparator	Cost (€)	QALY	Incremental cost (€)	Incremental QALY	ICER (cost/QALY)	Subset
Rx cohort	2,021,905.24	30,713.15				Dominant
No Rx cohort	14,536,501.25	14,745.26	-12,514,596.01	15,967.89	-783.74	Dominated

**Table 4:** results of Markov Simulation Model comparing cost-effectiveness of Harvoni compared to no treatment for female HCV patients in Seychelles

Comparator	Cost (€)	QALY	Inc. cost (€)	Inc. QALY	ICER (cost/QALY)	Subset
Rx cohort	2,046,465.72	37,244.88				Dominant
No Rx cohort	15,572,312.99	15,951.06	-13,525,847.27	21,293.82	-635.20	Dominated

**Figure 3:**
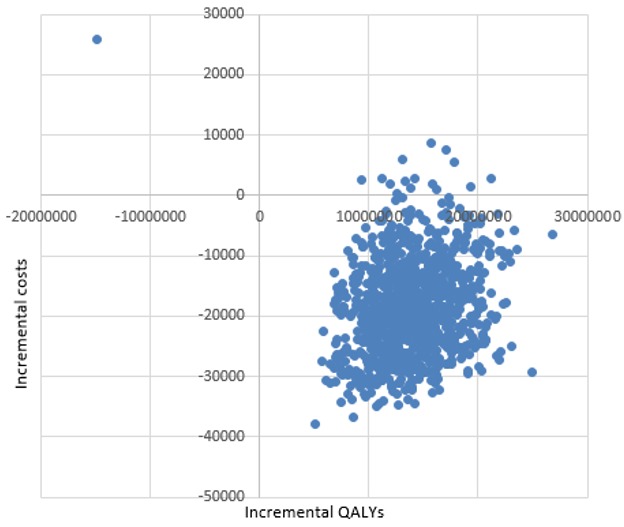
scatter plot of incremental cost-effectiveness of Harvoni (SOF/LDV) as compared to no treatment

**Figure 4:**
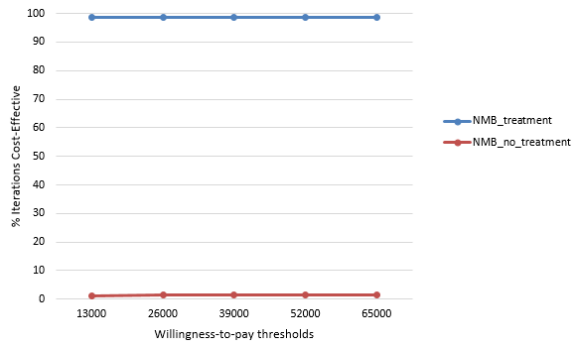
cost-effectiveness acceptability curve for the simulation

**Sensitivity analysis:** due to the lack of primary data from Seychelles, there is likely to be considerable uncertainty around the ICER estimates. This uncertainty is addressed in probabilistic sensitivity analysis (PSA) by varying input parameters according to a distribution, as shown in Table 5. The parameters used in this study were distributed. Transition probabilities and utility values ranged from 0 to 1, and so beta (β) distribution was used to determine their distribution. For the determination of distribution of costs, a gamma (γ) distribution was used. A simulation using 1000 trials was conducted in excel for the probabilistic sensitivity analysis (PSA). The PSA results can be presented on a cost-effectiveness plane, as shown in Figure 3. The X axis (horizontal axis) measures the incremental effect and the Y axis (vertical axis) measures the incremental costs. The north-west quadrant represents a situation whereby the new treatment is both, costlier and less effective, rendering it dominated by no treatment. The south-east quadrant represents a situation whereby the new treatment is more effective, being less costly and more effective than not treating hepatitis C. The north east and south west quadrants are less straightforward, showing a costlier and more effective, or a less costly but less effective situation, respectively. In these two situations, cost-effectiveness is decided according to whether the ICER is above the willingness to pay threshold. Figure 3 shows that in most of the simulations, the estimated ICER falls into the south-east quadrant. Hence, the new treatment with Harvoni is more effective and less costly than 'no treatment' option. Most of the uncertainty seems to come from the costs, given that the spread of data points is wider for costs (vertically) than for QALYs (horizontally). Figure 4 shows the cost-effectiveness acceptability curve (CEAC) depicting the percentage of the simulation that were cost-effective for Harvoni (Neuromuscular blockade (NMB) treatment), and for no treatment option (NMB treatment), at different willingness to pay thresholds. The different willingness to pay threshold presented are approximately one, two, three, and four times the GDP per capita of Seychelles in Euro, shown as 13000, 26000, 39000, 52000, and 65000, respectively. According to the CEAC, even at a lower WTP, there is still a high probability (>97%) that treatment will be cost-effective, compared with 'no treatment'.

**Table 5:** parameters used in PSA

Parameter	Deterministic	SE	Distribution
P_CHC_SVR	0.977	0.003	Beta
P_CHC_CC	0.115	0.04106	Beta
P_CC_DC	0.039	0.04106	Beta
P_CC_HCC	0.024	0.04106	Beta
P_DC_HCC	0.014	0.04106	Beta
P_CHC_Death	Based on age and gender	Based on age and gender	Beta
P_CC_Death	Based on age and gender	Based on age and gender	Beta
P_DC_Death	0.13	0.02	Beta
P_HCC_Death	0.43	0.06	Beta
U_SVR	0.79	0.12	Beta
U_CHC	0.74	0.03	Beta
U_CC	0.55	0.1	Beta
U_DC	0.45	0.06	Beta
U_HCC	0.45	0.06	Beta
C_treatment	1377.05	300	Gamma
C_CHC	256.67	60	Gamma
C_CC	926.79	200	Gamma
C_DC	2668.91	600	Gamma
C_HCC	2955.40	680	Gamma

P_: transition probability; U_: utility; C_: cost; SE: sampling error; SVR: sustained virologic response; CHC: chronic hepatitis C; CC: compensated cirrhosis; DC: decopensated cirrhosis; HCC: hepatocellular carcinoma

## Discussion

The results of this study show that treating hepatitis C in Seychelles with the direct-acting antiviral (DAAs) Harvoni (SOF/LDV) is cost-saving, as compared to no treatment, for a time horizon of 60 years. This result is robust, as demonstrated by PSA. The study has several strengths which support its credibility. Firstly, the country-specific data used from Seychelles were representative of the local situation. The simulated cohorts were representative of the cohort of patients with HCV infection in Seychelles at the end of 2016, sourced from DRSU of the Seychelles Public Health Authority. The costs data of the two treatment options were sourced from the Communicable Disease Control Unit CDCU budget report, the department responsible for follow-up and treatment of HCV patients. This improves the relevance of the results to the current Seychelles scenario. Secondly, the incorporated conditions are relevant to Seychelles, such as patient characteristics, treatment costs as per Seychelles budget report, and exclusion of liver transplantation which is not offered in Seychelles. This allows the result to be generalisable to Seychelles context. Limitations were mostly attributed to availability of disaggregated data from Seychelles. Firstly, the risk of re-infection was not considered in this model. No new infection entered the model over the years either. This may have underestimated the cost of treatment. However, the value of treatment through infections prevented was also not considered, underestimating the value of treatment in the same aspect. In other words, the ability for treatment of one person to decrease incidence by decreasing risk of infection to others is not valued here. Similarly, other studies reviewed in the literature did not capture this value. Secondly, the transition probabilities and utility values were from other countries. The EuroQol 5 dimensions questionnaire used to report utilities are contextual and involved extrapolation from a different setting to Seychelles. Using utility questionnaire related to Seychelles would have given a more accurate result which are representative of this model of HCV patients in the Seychelles context. Thirdly, a single efficacy estimate of Harvoni was used, from Alavian *et al.*, minimising its accuracy [12]. Ideally, a systematic review result of efficacy should be used. However, such publications are limited, given that Harvoni has only been introduced within the last few years. The model assumes that the genotypes present are sensitive to Harvoni, leading to the possibility of over-estimating the efficacy of the treatment.

Only direct medical costs were considered, disregarding societal costs and consequences. This may have led to undervaluation of the treatment, and its ability to decrease travel costs to health centres, and increase work production. Furthermore, the lack of a similar study in a comparable country to Seychelles, in terms of population size, patient profiles, health care system construct, and income per capita, makes it a challenge to validate the results. Seychelles is currently prioritising patients who are at low risk of re-infection for enrolment into the treatment programme [10]. Accessibility to treatment for those who are at higher risk of spreading the infection, such as people who currently inject drugs and prisoners, remain a challenge. The secondary data of HCV patients in Seychelles revealed that 90% were People who inject drugs (PWID) and 25% were diagnosed while serving a prison sentence [11]. Screening with subsequent treatment of high risk patient groups need to be encouraged to slow down further transmission rate and decrease incidence. Hopefully, these results will motivate the extension of treatment programme to target more PWID and prisoners as well. The cost of DAAs remain one of the main barriers of HCV treatment. Even if it has been proven that treatment is cost-effective, the initial cost of treatment is still high, particularly for a developing country. The results of this research show how the treatment is not only cost-effective, but cost saving. Treatment is an investment, from which benefits will be acquired decades later. It is, therefore, tactical to find ways to treat HCV before the complications set in. One way to dilute the high initial cost of treatment can be to spread it out over years. For example, an aim of treating minimum 200 cases per year may be more feasible than treating 1000 cases at one time. Policy makers can use these results to make evidenced-based decisions on what treatment policy will work best for Seychelles. It is noteworthy that from a politician's point of view, short-term benefits are more attractive than long term ones. Therefore, the initial higher cost of treatment, even after spreading it across a few years, remains a strong barrier to policy amendments towards treatment of all patients with hepatitis C. Increasing awareness amongst health workers and the public may be the bottom-up approach required to motivate policy makers to make decisions based on scientific evidence. The method of only treating patients at low risk of re-infection in Seychelles is arguably the least cost-effective method of treatment. The high risk of HCV transmission in people who inject drugs (PWID) means that their treatment not only decreases their risk of progressing to liver diseases, but also decreases the incidence of HCV infection in the general population. Treatment of PWID can also be a prevention programme, by decreasing risk of transmission of HCV from this person to others. One proposed way of reducing the risk of infection in PWID is to use the 'treat your friends' approach, proposed by Hellard *et al* [3]. The theory behind this is that most PWID only share needles with a small group of other injectors whom they trust [3]. Using this approach of treating the whole cluster of injectors at one time reduces risk of re-infection, as their risk is highly reliant on the HCV status of the people they share needles with [3].

## Conclusion

In conclusion, the findings of this study demonstrate that treatment of hepatitis C cases in Seychelles is cost-saving as opposed to no treatment. Additionally, it makes the WHO's goal to eliminate hepatitis C as a public health burden by 2030 an achievable one for Seychelles. These results make a strong case for expansion of the treatment programme in Seychelles to benefit all HCV patients, regardless of status of drug injection. Accompanying efficient prevention programmes, and an expended screening programme, the treatment has the ability to decrease HCV incidence.

### What is known about this topic

- The first all-oral HCV treatment, direct-acting antivirals (DAAs), were approved by the European Commission in January 2014;
- The first all-oral HCV treatment, direct-acting antivirals (DAAs), were approved by the European Commission in January 2014;
- The first all-oral HCV treatment, direct-acting antivirals (DAAs), were approved by the European Commission in January 2014.

### What this study adds

- Cost-effectiveness of treating hepatitis C in an sub-Saharan African country and in a Small Island Developing State (SIDS). pattern where non-camp resident patients admitted to the IPD, present with significantly increased mortality compared to camp resident patients might also indicate unaddressed health needs in the surrounding area.
